# Olfactory Dysfunction in Frontline Health Care Professionals During COVID-19 Pandemic in Brazil

**DOI:** 10.3389/fphys.2021.622987

**Published:** 2021-03-09

**Authors:** Mariana Ferreira Sbrana, Marco Aurélio Fornazieri, Alexandre Bruni-Cardoso, Vivian I. Avelino-Silva, Deborah Schechtman, Richard Louis Voegels, Bettina Malnic, Isaias Glezer, Fabio de Rezende Pinna

**Affiliations:** ^1^Department of Otorhinolaryngology, Faculdade de Medicina da Universidade de São Paulo, São Paulo, Brazil; ^2^Department of Clinical Surgery, Universidade Estadual de Londrina and Pontifical Catholic University of Paraná, Londrina, Brazil; ^3^Department of Biochemistry, Institute of Chemistry, Universidade de São Paulo, São Paulo, Brazil; ^4^Department of Infectious and Parasitic Diseases, Faculdade de Medicina da Universidade de São Paulo, São Paulo, Brazil; ^5^Department of Biochemistry, Escola Paulista de Medicina, Universidade Federal de São Paulo, São Paulo, Brazil

**Keywords:** coronavirus, COVID-19, olfaction disorders, respiratory tract infection, health care, sense of smell, SARS-CoV-2, anosmia

## Abstract

Upper respiratory viral infections can decrease the sense of smell either by inflammatory restriction of nasal airflow that carries the odorant molecules or through interference in olfactory sensory neuron function. During the coronavirus disease 2019 (COVID-19) pandemic, triggered by severe acute respiratory syndrome coronavirus 2 (SARS-CoV-2), worldwide reports of severe smell loss (anosmia/hyposmia) revealed a different type of olfactory dysfunction associated with respiratory virus infection. Since self-reported perception of smell is subjective and SARS-CoV-2 exposure is variable in the general population, we aimed to study a population that would be more homogeneously exposed to the virus. Here, we investigated the prevalence of olfactory loss in frontline health professionals diagnosed with COVID-19 in Brazil, one of the major epicenters of the disease. We also analyzed the rate of olfactory function recovery and the particular characteristics of olfactory deficit in this population. A widely disclosed cross-sectional online survey directed to health care workers was developed by a group of researchers to collect data concerning demographic information, general symptoms, otolaryngological symptoms, comorbidities, and COVID-19 test results. Of the 1,376 health professionals who completed the questionnaire, 795 (57.8%) were working directly with COVID-19 patients, either in intensive care units, emergency rooms, wards, outpatient clinics, or other areas. Five-hundred forty-one (39.3%) participants tested positive for SARS-CoV-2, and 509 (37%) were not tested. Prevalence of olfactory dysfunction in COVID-19-positive subjects was 83.9% (454 of 541) compared to 12.9% (42 of 326) of those who tested negative and to 14.9% (76 of 509) of those not tested. Olfactory dysfunction incidence was higher in those working in wards, emergency rooms, and intensive care units compared to professionals in outpatient clinics. In general, remission from olfactory symptoms was frequent by the time of responses. Taste disturbances were present in 74.1% of infected participants and were significantly associated with hyposmia. In conclusion, olfactory dysfunction is highly correlated with exposure to SARS-CoV-2 in health care professionals, and remission rates up to 2 weeks are high.

## Introduction

Clinical presentation of patients infected by severe acute respiratory syndrome coronavirus 2 (SARS-CoV-2) varies from asymptomatic infection to mild and severe systemic symptoms (Chan et al., 2020). Common symptoms include fever, myalgia, cough, and fatigue (Guan et al., 2020). Sore throat, nasal congestion, rhinorrhea, headache, and diarrhea have also been described (Chen et al., 2020; Huang et al., 2020). Since the beginning of the pandemic, an increasing number of patients have sought medical assistance reporting loss of smell (Hopkins and Kumar, 2020; Iran News, 2020) and, thereby, a number of studies have been conducted to analyze the prevalence and determinants of olfactory dysfunction in coronavirus disease 2019 (COVID-19) patients.

The studies published so far have shown a prevalence of loss of smell in SARS-CoV-2-infected patients ranging from 5.1 to 85.6% (Lechien et al., 2020; Mao et al., 2020; Menni et al., 2020; Parma et al., 2020; Spinato et al., 2020) and suggested that SARS-CoV-2-related anosmia/hyposmia may differ from that associated with other respiratory virus infections, affecting patients with no other upper respiratory tract symptoms (Gane et al., 2020).

The primary objective of this study was to investigate the prevalence of olfactory loss among frontline health professionals according to exposure to COVID-19 in Brazil, one of the major epicenters of the disease. As secondary objectives, we aimed to analyze the frequency of olfactory function recovery during the period of study and the particular characteristics of hyposmia/anosmia (relation to other nasal symptoms, duration, and recovery time) in this population.

## Materials and Methods

The current study was approved by the ethics committee of *Hospital das Clinicas of University of São Paulo, Brazil* (approval number: 4.047.527). All participants provided consent for participation through the electronic questionnaire platform.

### Subject Population

Health care workers were invited to answer an online questionnaire widely disclosed through social media, institutional mailing lists, regional professional councils, and radio. We excluded health professionals who did not inform a valid professional registration number, those who did not live in Brazil, those working in administrative jobs, those not in direct contact with patients, and those who reported contradictory answers related to olfactory/taste symptoms, for instance, answered “loss or reduction of smell” for one question and “I did not lose sense of smell” for a subsequent question (see Supplementary File for detailed survey questions).

COVID-19 epidemiological dataset (Brazilian cities) is publicly available at https://brasil.io.

### Clinical Outcomes

A cross-sectional study was carried out, and clinical data were collected from May 29 to July 8. By the time we started this work, we did not have standard questionnaires to evaluate olfactory and taste functions available in Portuguese. Therefore, participants answered an electronic nonstandard questionnaire^1^ developed by a group of researchers and based on previous studies that assessed olfactory complaints in COVID-19 patients. It consisted of 17 questions concerning demographic information (age, sex, e-mail, professional council number, state, occupation, area of professional practice, care for COVID-19 patients), general symptoms, ear/nose/throat (ENT) symptoms (including olfactory and gustatory symptoms), comorbidities that are known to impair smell function (such as chronic rhinosinusitis, neurodegenerative diseases, smoking, traumatic brain injury, stroke, epilepsy, brain tumor, the use of psychiatric or heart disease medications), and COVID-19 test results (the questionnaire is available as Supplementary Material).

### Statistical Analysis

Statistical analysis was performed using statistical software STATA 13.0 (StataCorp LP, College Station, TX, United States) and R software environment (v3.6 Linux version; using standard packages like ggplot, tidyverse, ggmap, sf, etc., in addition to easyalluvial and ComplexUpset) (Wickham, 2016; Koneswarakantha, 2020; Krassowski, 2020; R Core Team, 2020). Continuous data were described as mean and standard deviation or as median and interquartile range, and categorical data were described as percentages. Potential associations between categorical variables have been assessed through chi-square test. The risk of olfactory disturbances between different groups of health care professionals has been assessed through logistic regression analysis. The independent variables used in logistic regression analysis were age, sex, SARS-CoV-2 positivity, area of practice [ward, emergency room, intensive care unit (ICU), clinic], comorbidities, occupation, and nasal symptoms (obstruction, rhinorrhea, burning sensation). The Bonferroni approach was used to adjust the level of statistical significance in multiple hypothesis testing. A level of *p* < 0.05 was set to determine statistical significance.

Correlations between some survey answers were displayed through alluvial plots, which employ the style of Sankey diagram to visualize categorical data over multiple dimensions as flows (Rosvall and Bergstrom, 2010). Due to figure complexity of Venn and Euler diagrams when depicting group intersections, we employed UpSet as a visualization technique (Lex et al., 2014). Figures were assembled with Inkscape^2^ and converted to TIFF files with GIMP^3^.

## Results

### Subject Population

**FIGURE 1:**
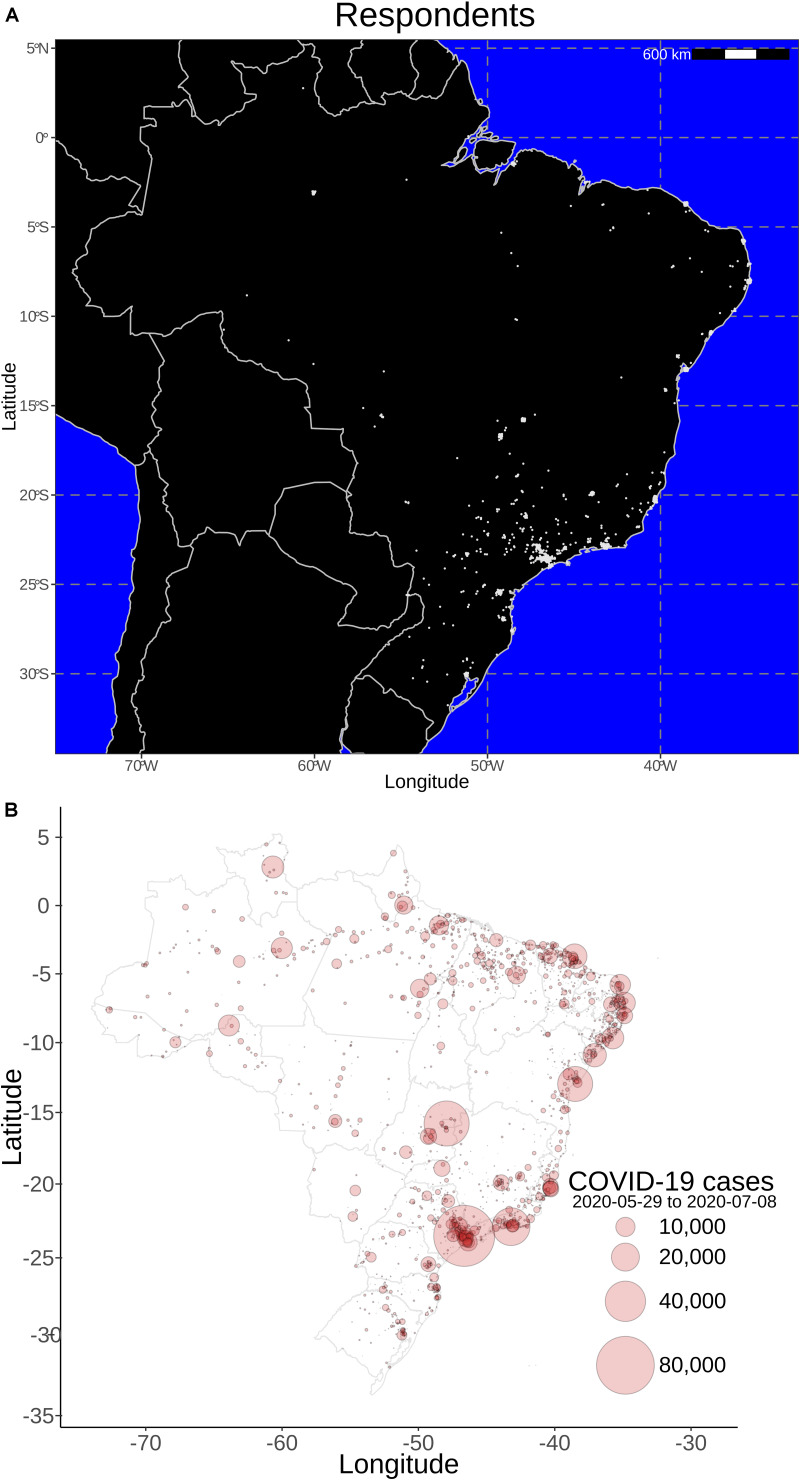
Geo-localization of online survey participants and epidemiological data. Approximate location of respondents who enrolled in the online survey **(A)**, indicating that the major coronavirus disease 2019 (COVID-19) hot spots in Brazil **(B)** are represented in the collected data (May 29, 2020 to July 8, 2020). Most of participants were from São Paulo, which has been the Brazilian epicenter.

Of the 1,499 individuals who answered the questionnaire, 123 were excluded because the access to the online survey was tracked to foreign regions (not Brazil) or because the respondent was identified as a non-health care professional, did not provide a valid professional council number, or provided contradictory answers. Therefore, 1,376 health professionals who completed the questionnaire were included in the analysis. Our survey collected nationwide answers concentrated in endemic Brazilian regions that registered a high number of COVID-19 cases in the period of data collection (from May 29, 2020, to July 8, 2020; Figure 1). Mean age of respondents was 38.9 ± 10.4 years old (range 20–80), 1,021 were females (74.2%). Seventy-eight professionals (5.6%) reported previous olfactory or taste disturbances. A total of 795 (57.8%) were working directly with COVID-19 patients, 356 (25.8%) participants worked in outpatient clinics, 228 (16.5%) in emergency rooms, 183 (13.3%) in ICUs, 109 (8%) in wards, and 495 (36%) in other areas. Table 1 summarizes clinical and demographic data of the health care professionals. As some questions were not mandatory to answer, there were participants who did not provide all information. These participants were not excluded because the missing questions would not compromise the analysis of our main objective.

**TABLE 1:** Health professionals’ clinical and demographic data.

	**Total**
	**(*N* = 1,376)**
Age, mean (SD), years	38.9 (10.4)
Female sex, No. (%)	1,021 (74.2)
**Comorbidities, No. (%)**	
Yes	299 (21.7)
No	1,020 (74.1)
No answer	57 (4.2)
**Professional practice local, No. (%)**	
Clinic	356 (25.8)
Emergency room	228 (16.5)
Intensive care unit	183 (13.3)
Ward	109 (8)
Other	495 (36)
No answer	5 (0.4)
**Working with COVID-19 patients? No. (%)**	
Yes	795 (57.8)
No	572 (41.5)
No answer	9 (0.7)
**COVID-19 tests, No. (%)**	
Positive	541 (39.3)
Negative	326 (23.7)
Non-tested	509 (37)
**Type of test, No. (% total) [% tested]**	
Only positive RT-PCR	427 (31) [49.2]
Only positive anti-COVID-19 IgG/IgM rapid test	82 (6) [9.5]
Positive RT-PCR and positive anti-COVID-19 IgG/IgM rapid test	24 (1.7) [2.8]
Positive RT-PCR and negative anti-COVID-19 IgG/IgM rapid test	3 (0.2) [0.3]
Negative RT-PCR and positive anti-COVID-19 IgG/IgM rapid test	5 (0.4) [0.6]
Only negative RT-PCR	106 (7.7) [12.2]
Only negative anti-COVID-19 IgG/IgM rapid test	188 (13.6) [21.7]
Negative RT-PCR and negative anti-COVID-19 IgG/IgMrapid test	32 (2.3) [3.7]
**Previous olfactory deficit? No. (%)**	
Yes	78 (5.7)
No	1,249 (90.7)
No answer	49 (3.6)

### Clinical Outcomes

Five-hundred forty-one (39.3%) health care professionals tested positive for SARS-CoV-2 infection. Prevalence of olfactory dysfunction in COVID-19-positive subjects was 83.9% (454 of 541) compared to 12.9% (42 of 326) of those who tested negative and to 14.3% (73 of 509) of those not tested (Pearson chi-square 407.2, *p* < 0.001). Supplementary Table 1 summarizes overall symptoms reported by survey respondents, and Supplementary Tables 2, 3 present general variables and other symptoms associated with hyposmia.

**FIGURE 2:**
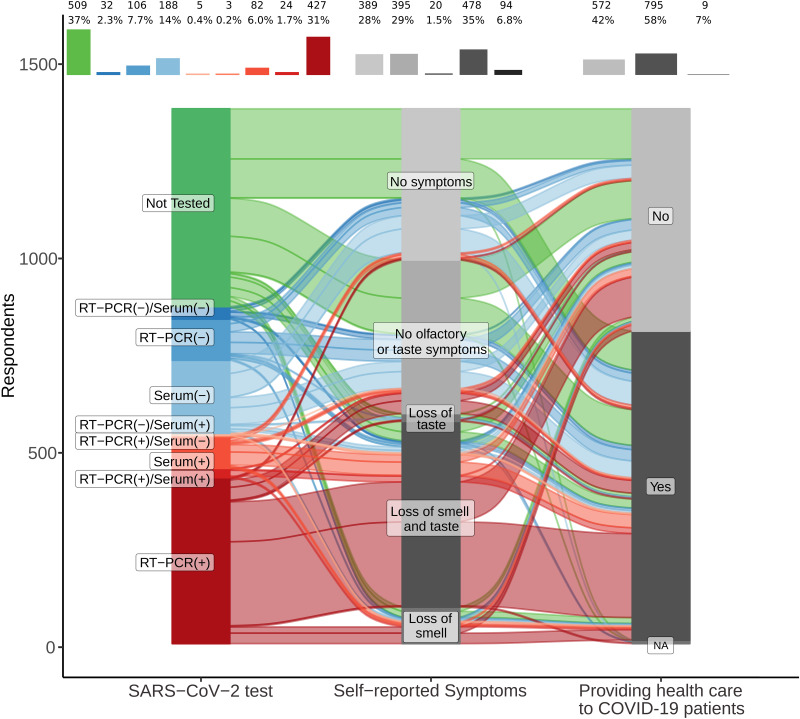
Loss of smell is associated with severe acute respiratory syndrome coronavirus 2 (SARS-CoV-2)-positive testing in professionals providing health care to coronavirus disease 2019 (COVID-19) patients. Alluvial diagram depicting SARS-CoV-2-positive respondents (shades of red) as the dominant proportion of those who self-reported loss of smell, combined or not with loss of taste. A higher proportion of participants tested positive in the group providing health care to COVID-19 patients. In contrast, SARS-CoV-2-negative (shades of blue) and not-tested (green) participants reported other symptoms or no symptoms more often than SARS-CoV-2-positive respondents. Frequency and percentages of each block are reported at the top of the diagram. Correlations between individual answers are represented as flows between one block to another (each block in the same column represents a different answer for the same question). The larger the width of a flow, the larger is the number/proportion of correlated answers from different questions. Questions (Q)/answer (A) pairs were (from left to right): (1) **Q** – Have you been tested for COVID-19? **A** – Yes or no (specify test method); (2) **Q** – Are you feeling or have you felt any of these symptoms in the last days? / **A** – Answers to loss or reduction of smell or taste were aggregated as “loss of smell,” “loss of smell and taste,” “loss of taste,” “no olfactory or taste symptoms,” “no symptoms”; (3) **Q** – Are you providing direct care to patients with COVID-19 diagnosis? / **A** – Yes or no. NA: not answered.

Direct care provision to COVID-19 patients tended to be associated with a positive test result: 64.8% of health care professionals working with SARS-COV-2-infected patients tested positive comparing to 58.4% of those who did not (Pearson chi-square 3.42, *p* = 0.064; proportions are depicted in Figure 2). While 38.7% of professionals providing health care to COVID-19 patients (795; 58% of respondents) tested positive for SARS-CoV-2 and reported loss of smell or taste (red color flow in Figure 2), only 25.7% of the group that did not provide health care to COVID-19 patients (572, 42% of respondents) tested positive for SARS-CoV-2 and showed these same symptoms. Only 2.9% of respondents providing health care to COVID-19 patients tested negative for coronavirus (blue color flows) and reported loss of smell or taste. Similarly, only 5.9% of the professionals who provided care to COVID-19 patients, but were not tested for SARS-CoV-2, reported hyposmia or hypogeusia. Professionals who tested negative or did not test for SARS-CoV-2 predominate in the subset that did not report olfactory or taste impairment (blocks identified as “No symptoms” or “No olfactory or taste symptoms”; Figure 2), independent of treating COVID-19 patients or not.

Prevalence of olfactory dysfunction was higher in professionals working in wards (61.5%), ICUs (60.1%), and emergency rooms (44.3%) compared to those working in outpatient clinics (22.3%), either positive for SARS-CoV-2 infection or not (Pearson chi-square 93.4, *p* < 0.001; Supplementary Figure 1). Physiotherapists were the professional category with the highest prevalence of olfactory dysfunction (59.6%), followed by nurses (54.1%), doctors (36.5%), and speech therapists (25.7%; Pearson chi-square 45.3, *p* < 0.001). Physiotherapists were also the category represented by a higher proportion of positive tests (Pearson chi-square 24.9, *p* < 0.001).

We performed a logistic regression analysis to evaluate the factors related to the risk of presenting smell loss, and we found an odds two times higher in subjects working in wards compared to professionals working in outpatient clinics [odds ratio (OR): 2.4; CI 95% 1.1–5.4, *p* = 0.03]. It was also higher for those working in the ICU (OR: 1.8; CI 95% 1–3.4, *p* = 0.049). We found no statistically significant association between olfactory loss and comorbidities, occupation, or providing direct care to SARS-CoV-2-infected patients (*p* > 0.05). Table 2 illustrates the risk of diminished smell perception according to the variables studied.

**TABLE 2:** Logistic regression for the variables related to diminished smell perception.

**Presence of olfactory dysfunction**	**Coef.**	**St. err.**	***t* value**	***p* value**	**[95% Conf. Interval]**		**Sig**
Positivity for COVID-19	23.28	4.164	17.60	0.000	16.398	33.059	***
Sex (male as ref.)	1.163	0.239	0.74	0.462	0.778	1.739	
Age	0.985	0.009	–1.72	0.086	0.968	1.002	*
**Area of practice**							
Clinic (ref.)	1	.	.	.	.	.	
Intensive Care Unit	1.841	0.572	1.97	0.049	1.002	3.384	**
Ward	2.424	1.001	2.14	0.032	1.079	5.446	**
Emergency room	1.552	0.418	1.63	0.103	0.915	2.633	
Other places	1.71	0.506	1.82	0.07	0.958	3.053	*
**Working with COVID-19 patients**	0.915	0.174	–0.47	0.641	0.63	1.329	
**Profession**							
Speech therapist (ref.)	1	.	.	.	.	.	
Nurse	1.423	0.512	0.98	0.328	0.702	2.881	
Physician	1.091	0.427	0.22	0.823	0.507	2.348	
Physiotherapist	1.41	0.794	0.61	0.542	0.467	4.253	
**Nasal symptoms**							
Coryza	1.393	0.28	1.65	0.099	0.94	2.065	*
Nasal obstruction	2.148	0.444	3.70	0.000	1.433	3.222	***
Nasal burning	4.212	0.981	6.18	0.000	2.669	6.648	***
Comorbidities	1.099	0.254	0.41	0.681	0.699	1.728	
Constant	0.095	0.057	–3.94	0.000	0.03	0.307	***

Mean dependent var		0.406	SD dependent var			0.491	
Pseudo r-squared		0.454	Number of obs			1,256	
Chi-square		770.696	Prob > chi^2^			0.000	
Akaike crit. (AIC)		957.882	Bayesian crit. (BIC)			1, 040.053	

***p < 0.01, **p < 0.05, *p < 0.1, ref. = reference.

**FIGURE 3:**
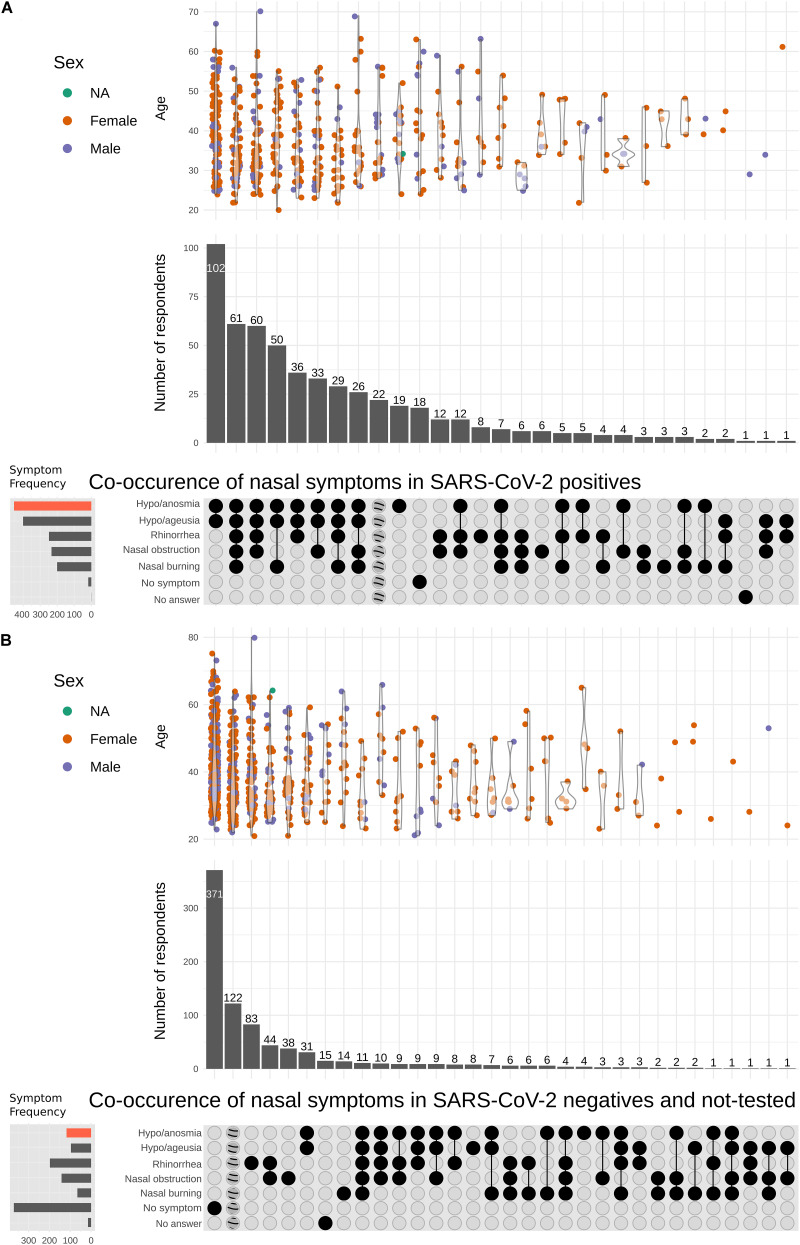
Nasal symptoms in severe acute respiratory syndrome coronavirus 2 (SARS-CoV-2)-infected participants. Co-occurrence of different nasal symptoms (rhinorrhea, nasal obstruction, and nasal burning sensation) in SARS-CoV-2-positive **(A)** and SARS-CoV-2-negative or not-tested respondents **(B)**. Age and sex are represented as violin plots for each symptom co-occurrence (top). UpSet plots depict the relationships between the symptom sets (bottom). The vertical bars represent the number of respondents who reported each one of the symptom co-occurrences. The horizontal bars shown to the left indicate the total frequencies of each individual symptom, with the red shade-filled bar denoting the sharp contrast between the frequency of hyposmia/anosmia symptoms in SARS-CoV-2-infected participants and in those who tested negative/not tested. Subjects who did not report any of the symptoms are identified as “No symptom.” Those who only reported symptoms unrelated to the nasal symptoms are identified by the dashed circles.

We also analyzed the association between nasal symptoms (rhinorrhea, obstruction, and burning sensation) and olfactory loss in all participants complaining of hyposmia (Figure 3 and Supplementary Figure 2). Thirty-one percent of subjects without nasal obstruction had olfactory loss, while 67.8% with nasal obstruction complained of olfactory deficit. When considering only patients with COVID-19, 80.8% of patients who did not report nasal obstruction had olfactory dysfunction. The proportion of subjects with nasal burning sensation and nasal obstruction were higher in those with olfactory loss (Pearson chi-squares 146.8 and 219.1, respectively; *p* < 0.001). Participants who presented nasal burning sensation and nasal obstruction had a higher risk of complaining of olfactory loss (OR: 4.2, CI 95% 2.7–6.6, *p* < 0.001 and OR: 2.1, CI 95% 1.4–3.2, *p* < 0.001, respectively) independent of the SARS-CoV-2 test result. While loss of smell and taste were the most frequent symptoms in the infected respondents, other symptoms were variably concurrent (Figure 3A and Supplementary Table 3). In SARS-CoV-2-negative or not-tested respondents, the absence of symptoms and non-nasal symptoms were the most frequent reports, followed by rhinorrhea and nasal obstruction without concurrent loss of smell (Figure 3B).

**FIGURE 4:**
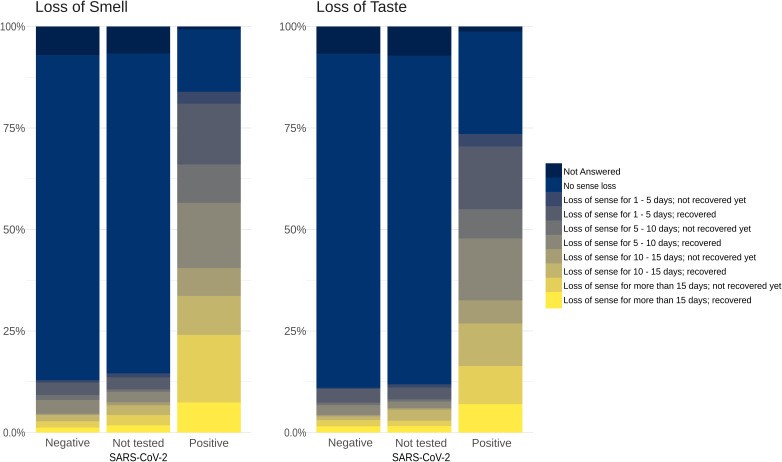
Duration and recovery of loss of smell and taste. Proportions based on the period of olfactory/taste function recovery are represented as stacked bar charts that depict the longer duration of loss of smell and loss of taste in severe acute respiratory syndrome coronavirus 2 (SARS-CoV-2)-positive participants. Color scale shows the duration of loss of sense and the remission status.

Loss of smell usually developed simultaneously with other COVID-19 symptoms, but in 7.5% of cases, olfactory dysfunction was the first disease indicator (Table 3). Altogether, remission from olfactory symptoms was frequent in positive subjects by the time of responses (57.2%), and in 48.4%, it happened in the first 2 weeks. When considering participants who tested negative or who did not test for SARS-CoV-2 infection and who presented loss of smell, 66.9% had recovered the olfactory function by the time they answered the questionnaire, a percentage of 9.7% higher than that of infected participants (Figure 4).

**TABLE 3:** Moment of occurrence of olfactory loss in COVID-19-positive participants and COVID-19-negative and non-tested participants.

**Moment of smell lost compared to others symptoms**	**COVID-19 positive *N* (%)**	**COVID-19 negative or non-tested *N* (%)**
Before others symptoms	34 (7.5)	21 (17.8)
Concurrent to others symptoms	239 (52.6)	60 (50.8)
After other symptoms	177 (39)	34 (28.8)

Taste dysfunction was present in 74.1% of infected subjects (401 of 541) compared to 11% (36 of 326) of noninfected individuals and was significantly associated with olfactory disturbances (Pearson chi-square 951.4, *p* < 0.001). By the time they answered the questionnaire, gustatory function had recovered in 65.3% of COVID-19-positive participants (Figure 4).

## Discussion

This study analyzed smell and taste disorders in a large sample of frontline health care workers, a population widely and homogeneously exposed to SARS-CoV-2 infection. We found a high prevalence of chemosensory disorders among professionals with confirmed COVID-19 in Brazil, in accordance with previous studies (Bagheri et al., 2020; Lechien et al., 2020; Menni et al., 2020; Neto et al., 2020; Parma et al., 2020; Spinato et al., 2020), which was significantly higher than those who tested negative. Interestingly, these symptoms were not associated with previous comorbidities known to impair olfactory function, such as chronic rhinosinusitis, neurodegenerative diseases, smoking, traumatic brain injury, stroke, or the use of psychiatric or heart disease medications. Chemosensory dysfunction occurred mostly concomitant to other COVID-19 symptoms and was the first sign of disease in 7.5% of participants. Olfactory disturbances were not more common in those providing direct care to infected patients. It suggests that to be exposed to a higher viral load does not increase the risk of olfactory impairment in health care professionals.

Almost half of all COVID-19-positive participants had recovered their olfactory function within 2 weeks of onset of symptoms. This early recovery was also observed by other authors (Hopkins et al., 2020; Lechien et al., 2020; Neto et al., 2020) and supports the hypothesis that the olfactory impairment is a transient symptom of COVID-19 patients. In fact, it has been demonstrated in animal studies that olfactory epithelium appeared intact at 7 days after SARS-CoV-2 intranasal inoculation (Zhang et al., 2020).

It is noteworthy that participants with olfactory complaints who were not tested or the ones who tested negative presented a recovery rate 9.7% higher than COVID-19-positive subjects by the time they answered the questionnaire (66.9 vs. 57.2%). This is an indication that, even though the mechanism is probably reversible, the degree of damage to the olfactory neuroepithelium caused by SARS-CoV-2 might be worse than that caused by other upper respiratory viruses; alternatively, the recovery of neuroepithelium damage following COVID-19 may take longer, increasing the risk of prolonged olfactory dysfunction. This long-lasting smell dysfunction was also documented by other authors, through objective measurement of olfactory function, even after clinical recovery and nasopharyngeal virologic clearance (Chung et al., 2020). A possible explanation might be SARS-CoV-2 infection of neuron progenitor cells, as suggested by Zhang et al. (2020), could impair olfactory sensory neuron regeneration.

In this study, we did not assess overall symptom severity because we tried to simplify the survey to completion in 3 min on average as a way to enlarge the study sample. We also did not ask participants about the need for hospitalization during the course of COVID-19, and since it was a self-reported questionnaire and we did not have access to participants’ medical records, we could not evaluate overall disease severity. However, if we observe the reported symptoms, only 91 of the infected respondents (16.8%) complained of dyspnea, chest pain, or difficulty to breath, which could suggest severe disease and may indicate that our sample is composed mostly of mild cases. Some authors suggested that, in mild-to-moderate cases, chemosensory dysfunction is more prevalent than in severe cases for which hospitalization is needed (Koneswarakantha, 2020). In fact, surveys including hospitalized patients show a lower prevalence of olfactory dysfunction, varying from 5 to 49% (Beltran-Corbelini et al., 2020; Giacomelli et al., 2020; Mao et al., 2020; Patel et al., 2020). Liu Y. et al. (2020) demonstrated that the mean viral load of patients with severe disease is higher than that of mild cases. However, when patients were stratified according to the day of disease onset, severe cases had lower nasopharyngeal viral loads in the first 12 days after disease onset than corresponding mildly symptomatic patients (Liu Y. et al., 2020). It is possible that, by the time nasopharyngeal viral loads increased in these patients, enhancing inflammatory response in upper airways, the respiratory distress overlaps with the olfactory symptoms so that they end up being neglected. Alternatively, nasopharyngeal and pulmonary/systemic viral replication and epithelial damage may occur independently.

It is of interest that smell loss was found in more than 80% of COVID-19 health professionals without nasal obstruction. This fact supports the hypothesis that olfactory impairment might be associated with an inflammatory response in olfactory neuroepithelium triggered by the virus or by cellular death secondary to SARS-CoV-2 infection. Both the respiratory epithelium and the olfactory neuroepithelium express angiotensin-converting enzyme 2 (ACE2) receptors, which are used by SARS-CoV-2 to enter cells (Brann et al., 2020; Hoffman et al., 2020; Liu M. et al., 2020; Sungnak et al., 2020). It is possible that the infection of supporting cells, which present the highest levels of ACE2 expression in the olfactory neuroepithelium (Fodoulian et al., 2020), triggers an inflammatory response that could either impair cellular signaling or cause death of the supporting cells, leading to loss of olfactory function (Glezer et al., 2020).

Another possible mechanism that could explain the presence of olfactory dysfunction in the absence of nasal obstruction is a cerebral involvement through virus dissemination from systemic circulation or from the cribriform plate (Baig et al., 2020; Zhou et al., 2020). Although olfactory neurons do not express ACE2 receptors, olfactory bulb vascular cells, glial cells, and brain neurons do, and these cells could be implicated in the pathogenesis of the disease (Alenina and Bader, 2019; Zhou et al., 2020). It has already been demonstrated that SARS-CoV, which is structurally similar to SARS-CoV-2 and uses the same ACE2 receptor, leads to neuronal death and invades the central nervous system after intranasal inoculation (Netland et al., 2008). Moreover, animal studies demonstrated that rodent coronaviruses invade the olfactory bulb even though olfactory neurons do not express their main receptor (Youngentob et al., 2001). It was also demonstrated that SARS-CoV-2 infects both mature and immature olfactory sensory neurons in hamsters after intranasal inoculation (Zhang et al., 2020). Kirschbaum et al. (2020) described autopsy findings of two patients with SARS-CoV-2 infection with olfactory neuropathy suggestive of axonal damage. However, studies with larger samples are necessary to confirm these theories in humans.

We found a statistically significant association between olfactory impairment and nose burning sensation in both positive and negative participants complaining of hyposmia. The sense of smell in humans is mostly mediated by cranial nerve I (CN I), responsible for odor sensation, through activation of olfactory neurons present in the olfactory epithelium (Doty and Mishra, 2001). The intranasal trigeminal nerve endings can also detect chemical stimuli and are typically activated by irritant stimuli, leading to varied sensations such as burning, cooling, and pungent sensations (Silver and Finger, 2009; Viana, 2011). There is evidence that the olfactory and trigeminal systems can interact and regulate each other, and it has been proposed that olfactory loss would lead to increased trigeminal sensitivity (Frasnelli and Hummel, 2007). This could explain the association between burning sensation and olfactory loss in the studied subjects, which appears to be independent of SARS-CoV-2 infection.

Our study has some limitations such as the fact that it was a self-reported electronic questionnaire, therefore subjects diagnosed with COVID-19 and who presented olfactory and gustatory disturbances are more inclined to voluntarily participate than those who did not. This could explain the high percentage of infected subjects (39.3%), the female predominance, and it could be partially responsible of the high prevalence of olfactory and taste disturbances. It has been demonstrated that women outperform men in olfactory tests (Doty et al., 1984), and therefore, they could be more sensitive to identify olfactory function deficits and, thus, more inclined to participate in surveys. This preponderance was also found in other online surveys (Bagheri et al., 2020; Hopkins et al., 2020; Lechien et al., 2020), despite the fact that COVID-19 has been suggested to be more prevalent in males (Hu et al., 2020; Remuzzi and Remuzzi, 2020). Giacomelli et al. (2020) performed a cross-sectional study in hospitalized patients with oral interviews and also found a female predominance with chemosensory disturbances even in a sample with a male majority. Besides that, it is possible that, once SARS-CoV-2 infection has a higher morbidity and mortality in males (Guo et al., 2020), and chemosensory disturbances have been associated with mild-to-moderate cases, this group would be less likely to report olfactory or taste dysfunctions.

As it was self-reported, there was no objective measurement of olfactory or gustatory functions. Research shows that self-report measures of smell are specific but not sensitive, and a considerable proportion of people do not recognize the loss of olfactory function (Adams et al., 2017). However, despite the possible lack of sensitivity, we found a large prevalence of chemosensory disturbances.

Another possible limitation was the use of a nonstandard questionnaire. However, by the time we started this work, we did not have standard questionnaires to evaluate olfactory and taste functions available in Portuguese.

Alongside the abovementioned conclusions, it is important to note the high rate of non-tested subjects (37%), particularly between participants who did not provide direct care to COVID-19 patients (46.6%). When considering the non-tested group, 14.9% of respondents presented olfactory loss—what could indicate the first sign of disease. However, since there were no other symptoms, these individuals were not tested. Therefore, it is possible that the prevalence of chemosensory disturbances would be even higher in the study sample.

## Conclusion

COVID-19 is associated with olfactory and gustatory disturbances, and this dysfunction occurs even in the absence of nose obstruction. It usually develops concurrent to other symptoms, but 7.5% of infected people can present it as the first sign of disease. Chemosensory impairment appears to have a good prognosis, and almost half of individuals presenting loss of smell recover olfactory function in the first 2 weeks of symptom onset, albeit a small proportion may maintain it for a longer period of time. However, when compared to non-tested and to SARS-CoV-2-negative subjects, COVID-19 participants had a lower rate of recovery of smell function during the period of study.

## Data Availability Statement

The raw data supporting the conclusions of this article will be made available by the authors, without undue reservation.

## Ethics Statement

The studies involving human participants were reviewed and approved by Ethics committee of Hospital das Clinicas of University of São Paulo, Brazil (approval number: 4.047.527). The patients/participants provided their written informed consent to participate in this study.

## Author Contributions

MS, AB-C, DS, BM, IG, and FR conceptualized and designed the study and developed the questionnaire. MS, IG, VA-S, and FR conducted the data collection. MF and IG conducted the analyses. MS drafted the initial manuscript. MS, MF, VA-S, DS, BM, IG, RV, and FR revised the manuscript and approved the final manuscript as submitted. All authors contributed to the article and approved the submitted version.

## Conflict of Interest

The authors declare that the research was conducted in the absence of any commercial or financial relationships that could be construed as a potential conflict of interest.

## Footnotes

1 https://pt.surveymonkey.com/r/ANOSMIACOVIDSAUDE

2 inkscape.org

3 gimp.org
