# Supplementary material for: Olfactory Dysfunction in Frontline Health Care Professionals During COVID-19 Pandemic in Brazil
Source: Front Physiol. 2021 Mar 9;12:622987. doi: 10.3389/fphys.2021.622987 (PMC7985267; doi:10.3389/fphys.2021.622987)
Supplement: Supplementary file 1 [file Table_1.pdf]

Olfactory Dysfunction in Frontline Health Care Professionals during COVID-19 Pandemic in Brazil

Mariana Ferreira Sbrana*[1], Marco Aurélio Fornazieri[2], Alexandre Bruni-Cardoso[3], Vivian I. Avelino-Silva[4], Deborah Schechtman[3], Richard Louis Voegels[1], Bettina Malnic[3], Isaias Glezer*[5], Fabio de Rezende Pinna[1]

[1]Department of Otorhinolaryngology, Faculdade de Medicina da Universidade de São Paulo, São Paulo, Brazil.
[2]Departament of Clinical Surgery, Universidade Estadual de Londrina and Pontifical Catholic University of Paraná, Londrina, Brazil.
[3]Departament of Biochemistry, Institute of Chemistry, Universidade de São Paulo, São Paulo, Brazil.
[4]Department of Infectious and Parasitic Diseases, Faculdade de Medicina da Universidade de São Paulo, São Paulo, Brazil.

[5]Departament of Biochemistry, UNIFESP, Escola Paulista de Medicina, Universidade Federal de São Paulo, São Paulo, Brazil.

Supplementary Tables and Figures

Table 1 – Overall symptoms reported by survey respondents

Table 2 – General variables associated with hyposmia self-reported by healthcare professionals.

Table 3 – Co-occurrence of hyposmia with other symptoms in respondents that tested for SARS-CoV-2 infection.

Figure S1 – Proportion of loss of sense of smell in healthcare professionals according to profession and working areas.

Figure S2 - Nasal symptoms in participants tested negative for SARS-CoV-2 infection compared to not-tested respondents.

Characteristic	N = 1,376[1]
Test	
Negative	326 (24%)
Not tested	509 (37%)
Positive	541 (39%)
Hypo/anosmia	572 (42%)
Hypo/ageusia	498 (36%)
Rhinorrhea	447 (32%)
Nasal obstruction	376 (27%)
Fatigue	421 (31%)
Sore throat	352 (26%)
Dry cough	355 (26%)
Nasal burning	267 (19%)
Other	207 (15%)
Abdominal pain	187 (14%)
Fever	218 (16%)
Nausea/vomiting	121 (8.8%)
Mucus cough	59 (4.3%)
Short breath	120 (8.7%)
Muscle pain	454 (33%)
no_Symptom	389 (28%)
NoAnswer	16 (1.2%)

[1] Statistics presented: n (%)

Table 1 – Overall symptoms reported by survey respondents

Variable	Hypo/anosmia			SARS-CoV-2 Test		
	**Overall**, N = 1,376[†]	**Absent**, N = 804[†]	**Present**, N = 572[†]	**Negative**, N = 326[†]	**Not tested**, N = 509[†]	**Positive**, N = 541[†]
**Age**	37 (31, 45)	38 (32, 47)	36 (30, 44)	39 (32, 47)	37 (31, 47)	36 (30, 44)
**Gender**						
	2 (0.1%)	1 (0.1%)	1 (0.2%)	0 (0%)	1 (0.2%)	1 (0.2%)
Female	1,021 (74%)	583 (73%)	438 (77%)	235 (72%)	384 (75%)	402 (74%)
Male	353 (26%)	220 (27%)	133 (23%)	91 (28%)	124 (24%)	138 (26%)
**Profession**						
Nurse	305 (22%)	140 (17%)	165 (29%)	62 (19%)	96 (19%)	147 (27%)
Other	111 (8.1%)	54 (6.7%)	57 (10.0%)	17 (5.2%)	40 (7.9%)	54 (10.0%)
Physician	808 (59%)	513 (64%)	295 (52%)	227 (70%)	283 (56%)	298 (55%)
Physiotherapist	47 (3.4%)	19 (2.4%)	28 (4.9%)	3 (0.9%)	17 (3.3%)	27 (5.0%)
Speech therapist	105 (7.6%)	78 (9.7%)	27 (4.7%)	17 (5.2%)	73 (14%)	15 (2.8%)
**Area**						
Clinic	356 (26%)	273 (34%)	83 (15%)	106 (33%)	166 (33%)	84 (16%)
Emergency Room	228 (17%)	127 (16%)	101 (18%)	57 (17%)	70 (14%)	101 (19%)
ICU	183 (13%)	73 (9.1%)	110 (19%)	37 (11%)	32 (6.3%)	114 (21%)
Other places	500 (36%)	289 (36%)	211 (37%)	103 (32%)	215 (42%)	182 (34%)
Ward	109 (7.9%)	42 (5.2%)	67 (12%)	23 (7.1%)	26 (5.1%)	60 (11%)
**Providing care to COVID-19 patients**						
	9 (0.7%)	4 (0.5%)	5 (0.9%)	3 (0.9%)	3 (0.6%)	3 (0.6%)
No	572 (42%)	368 (46%)	204 (36%)	127 (39%)	267 (52%)	178 (33%)
Yes	795 (58%)	432 (54%)	363 (63%)	196 (60%)	239 (47%)	360 (67%)
**Comorbidities**						
Absent	1,157 (84%)	685 (85%)	472 (83%)	268 (82%)	436 (86%)	453 (84%)
Present	219 (16%)	119 (15%)	100 (17%)	58 (18%)	73 (14%)	88 (16%)
**Smoke or smoked**						
No or not informed	1,284 (93%)	752 (94%)	532 (93%)	303 (93%)	468 (92%)	513 (95%)
Yes	92 (6.7%)	52 (6.5%)	40 (7.0%)	23 (7.1%)	41 (8.1%)	28 (5.2%)
**Sinusitis**						
Absent	1,254 (91%)	738 (92%)	516 (90%)	289 (89%)	474 (93%)	491 (91%)
Present	122 (8.9%)	66 (8.2%)	56 (9.8%)	37 (11%)	35 (6.9%)	50 (9.2%)
**Epilepsy**						
Absent	1,368 (99%)	796 (99%)	572 (100%)	323 (99%)	504 (99%)	541 (100%)
Present	8 (0.6%)	8 (1.0%)	0 (0%)	3 (0.9%)	5 (1.0%)	0 (0%)
**Neurodegenerative disease**						
Absent	1,374 (100%)	802 (100%)	572 (100%)	326 (100%)	507 (100%)	541 (100%)
Present	2 (0.1%)	2 (0.2%)	0 (0%)	0 (0%)	2 (0.4%)	0 (0%)
**Heart or psychiatric treatment**						
Absent	1,270 (92%)	751 (93%)	519 (91%)	302 (93%)	471 (93%)	497 (92%)
Present	106 (7.7%)	53 (6.6%)	53 (9.3%)	24 (7.4%)	38 (7.5%)	44 (8.1%)
**Test**						
Negative	326 (24%)	284 (35%)	42 (7.3%)			
Not tested	509 (37%)	433 (54%)	76 (13%)			
Positive	541 (39%)	87 (11%)	454 (79%)			
**Hypo/anosmia**						
Absent				284 (87%)	433 (85%)	87 (16%)
Present				42 (13%)	76 (15%)	454 (84%)

[†] Median (IQR) or Frequency (%)

Table 2 – General variables associated with hyposmia self-reported by healthcare professionals.

Variable	Hypo/anosmia			SARS-CoV-2 Test		
	**Overall**, N = 1,376[†]	**Absent**, N = 804[†]	**Present**, N = 572[†]	**Negative**, N = 326[†]	**Not tested**, N = 509[†]	**Positive**, N = 541[†]
**Hypo/ageusia**						
Absent	878 (64%)	784 (98%)	94 (16%)	290 (89%)	448 (88%)	140 (26%)
Present	498 (36%)	20 (2.5%)	478 (84%)	36 (11%)	61 (12%)	401 (74%)
**Rhinorrhea**						
Absent	929 (68%)	627 (78%)	302 (53%)	242 (74%)	395 (78%)	292 (54%)
Present	447 (32%)	177 (22%)	270 (47%)	84 (26%)	114 (22%)	249 (46%)
**Nasal obstruction**						
Absent	1,000 (73%)	683 (85%)	317 (55%)	273 (84%)	420 (83%)	307 (57%)
Present	376 (27%)	121 (15%)	255 (45%)	53 (16%)	89 (17%)	234 (43%)
**Fatigue**						
Absent	955 (69%)	681 (85%)	274 (48%)	277 (85%)	410 (81%)	268 (50%)
Present	421 (31%)	123 (15%)	298 (52%)	49 (15%)	99 (19%)	273 (50%)
**Sore throat**						
Absent	1,024 (74%)	666 (83%)	358 (63%)	264 (81%)	427 (84%)	333 (62%)
Present	352 (26%)	138 (17%)	214 (37%)	62 (19%)	82 (16%)	208 (38%)
**Dry cough**						
Absent	1,021 (74%)	697 (87%)	324 (57%)	270 (83%)	450 (88%)	301 (56%)
Present	355 (26%)	107 (13%)	248 (43%)	56 (17%)	59 (12%)	240 (44%)
**Nasal burning**						
Absent	1,109 (81%)	755 (94%)	354 (62%)	299 (92%)	470 (92%)	340 (63%)
Present	267 (19%)	49 (6.1%)	218 (38%)	27 (8.3%)	39 (7.7%)	201 (37%)
**Other**						
Absent	1,169 (85%)	733 (91%)	436 (76%)	297 (91%)	466 (92%)	406 (75%)
Present	207 (15%)	71 (8.8%)	136 (24%)	29 (8.9%)	43 (8.4%)	135 (25%)
**Abdominal pain**						
Absent	1,189 (86%)	731 (91%)	458 (80%)	302 (93%)	460 (90%)	427 (79%)
Present	187 (14%)	73 (9.1%)	114 (20%)	24 (7.4%)	49 (9.6%)	114 (21%)
**Fever**						
Absent	1,158 (84%)	767 (95%)	391 (68%)	313 (96%)	485 (95%)	360 (67%)
Present	218 (16%)	37 (4.6%)	181 (32%)	13 (4.0%)	24 (4.7%)	181 (33%)
**Nausea/vomiting**						
Absent	1,255 (91%)	776 (97%)	479 (84%)	319 (98%)	492 (97%)	444 (82%)
Present	121 (8.8%)	28 (3.5%)	93 (16%)	7 (2.1%)	17 (3.3%)	97 (18%)
**Mucus cough**						
Absent	1,317 (96%)	788 (98%)	529 (92%)	320 (98%)	497 (98%)	500 (92%)
Present	59 (4.3%)	16 (2.0%)	43 (7.5%)	6 (1.8%)	12 (2.4%)	41 (7.6%)
**Short breath**						
Absent	1,256 (91%)	772 (96%)	484 (85%)	310 (95%)	485 (95%)	461 (85%)
Present	120 (8.7%)	32 (4.0%)	88 (15%)	16 (4.9%)	24 (4.7%)	80 (15%)
**Muscle pain**						
Absent	922 (67%)	681 (85%)	241 (42%)	268 (82%)	427 (84%)	227 (42%)
Present	454 (33%)	123 (15%)	331 (58%)	58 (18%)	82 (16%)	314 (58%)
**No Symptom**						
Absent	987 (72%)	415 (52%)	572 (100%)	185 (57%)	279 (55%)	523 (97%)
Present	389 (28%)	389 (48%)	0 (0%)	141 (43%)	230 (45%)	18 (3.3%)
**No Answer**						
Absent	1,360 (99%)	788 (98%)	572 (100%)	319 (98%)	501 (98%)	540 (100%)
Present	16 (1.2%)	16 (2.0%)	0 (0%)	7 (2.1%)	8 (1.6%)	1 (0.2%)
**Test**						
Negative	326 (24%)	284 (35%)	42 (7.3%)			
Not tested	509 (37%)	433 (54%)	76 (13%)			
Positive	541 (39%)	87 (11%)	454 (79%)			
**Hypo/anosmia**						
Absent				284 (87%)	433 (85%)	87 (16%)
Present				42 (13%)	76 (15%)	454 (84%)

[†] Median (IQR) or Frequency (%)

Table 3 – Co-occurrence of hyposmia with other symptoms in respondents that tested for SARS-CoV-2 infection.

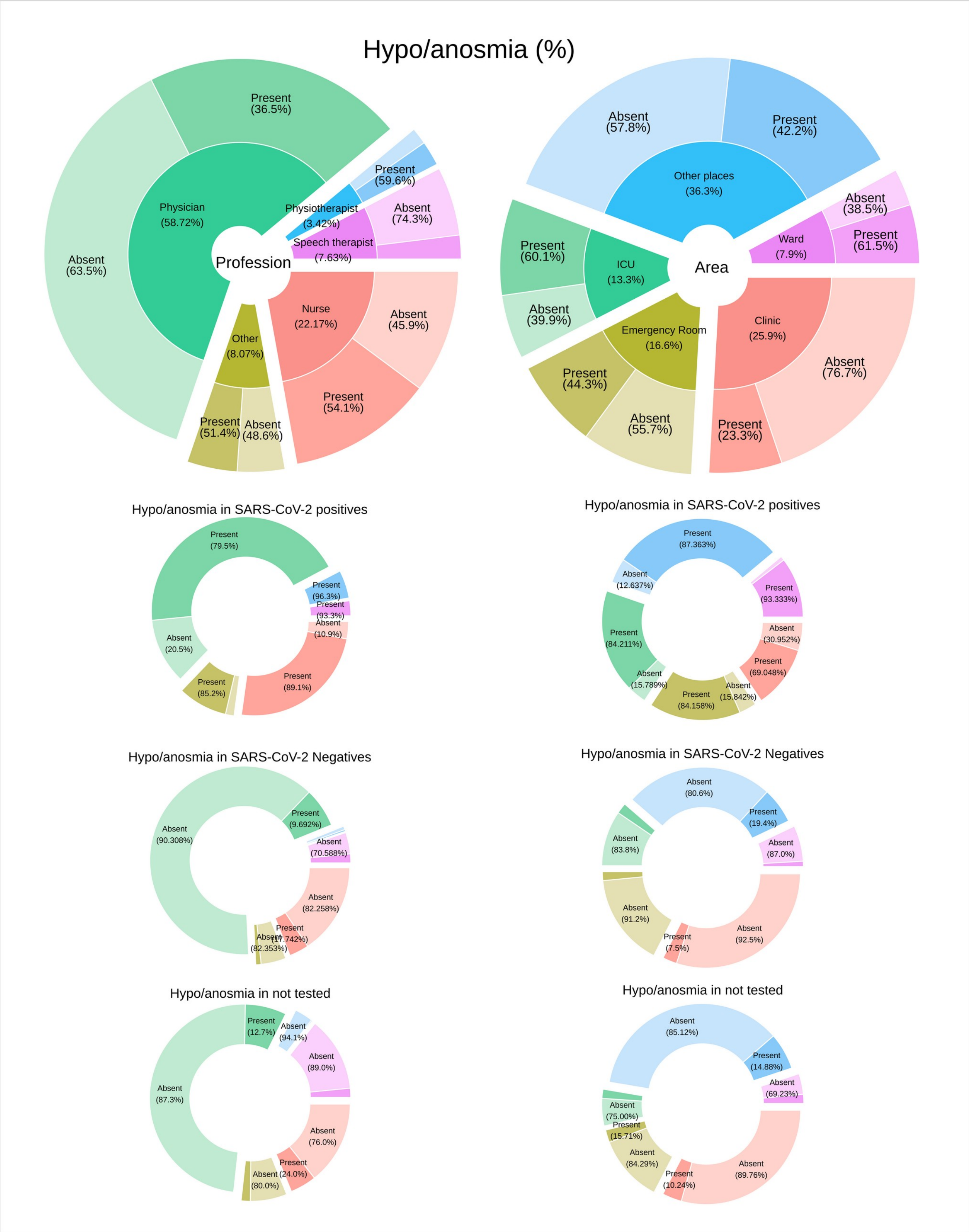

Figure S1 – Proportion of loss of sense of smell in healthcare professionals according to profession and working areas. Color-coded data segregated by professional (left) or working area (right).

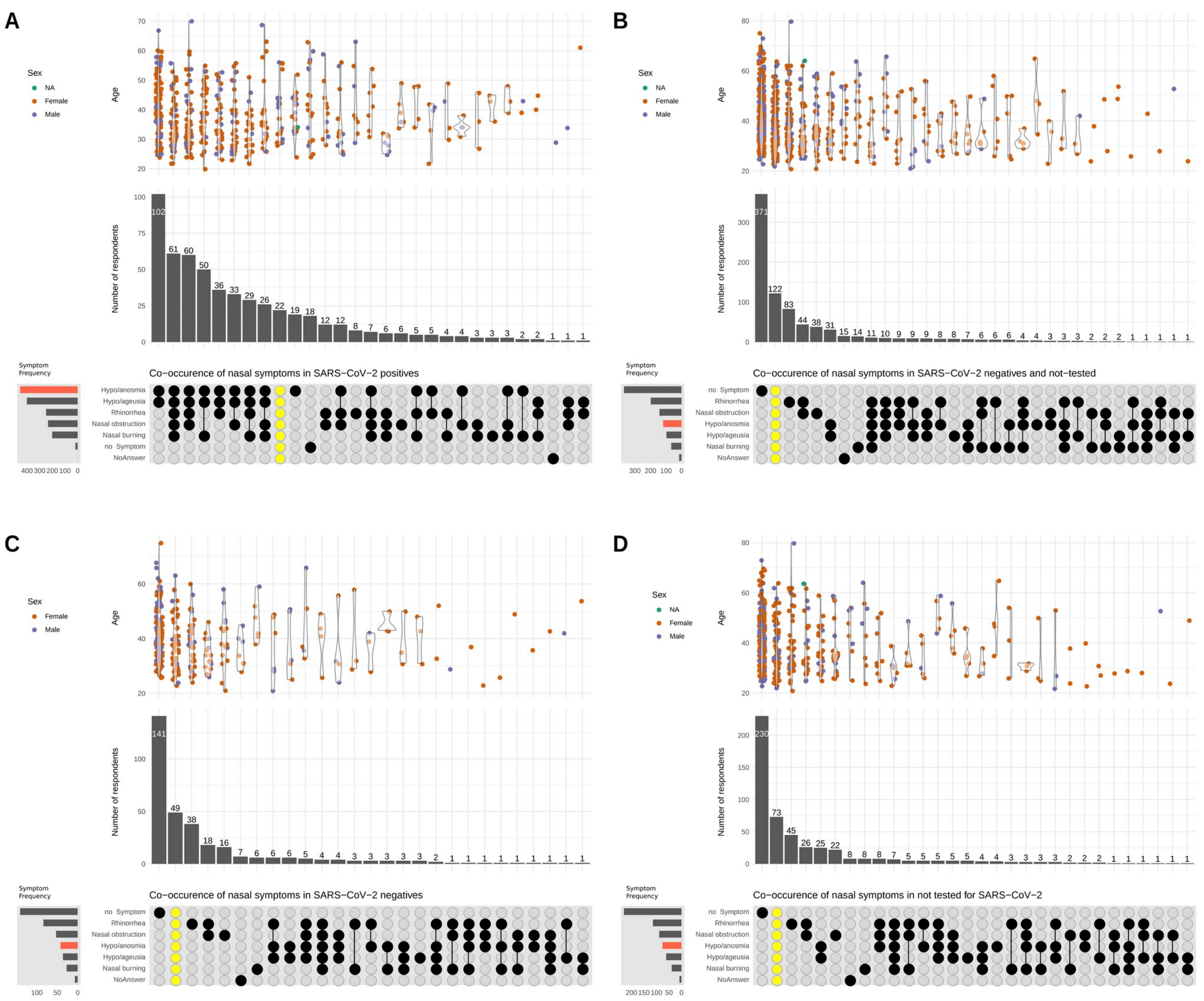

Figure S2 - Nasal symptoms in participants tested negative for SARS-CoV-2 infection compared to not-tested respondents. UpSet plots show co-occurrence of nasal symptoms (rhinorrhea, nasal obstruction and nasal burning sensation) in SARS-CoV-2-positive (A), SARS-CoV-2-negative and not-tested (B), only negatives (C), or only not-tested respondents (D). UpSet plots depict the relationships between symptom sets. Vertical bars on top of connected circles (symptoms) represent frequency of (co-)occurrence (i.e. set intersections) of nasal symptoms reported by respondents. Horizontal bars represent each symptom set total, i.e. total frequency of the specified symptom (set size). Age and sex are represented as violin plots for each symptom co-occurrence. Subjects that report no symptom occurrence are identified as “No symptom”, and those who did not provide symptom information are identified as “No answer”. Respondents that only reported other symptoms, not classified as ‘nasal’, are represented by the vertical column on top of yellow-filled circles.
