# Supplementary material for: Olfactory Dysfunction in Frontline Health Care Professionals During COVID-19 Pandemic in Brazil
Source: Front Physiol. 2021 Mar 9;12:622987. doi: 10.3389/fphys.2021.622987 (PMC7985267; doi:10.3389/fphys.2021.622987)
Supplement: Supplementary file 2 [file Table_2.pdf]

Olfactory Dysfunction in Frontline Health Care Professionals during COVID-19 Pandemic in Brazil

1 Suplemmentary text

Electronic questionnaire about Anosmia and COVID-19

1) Sex:
   - Female
   - Male
   - Other

2) Age:

3) Email:

4) Information about professional council:
   - Initials:

5) State:

6) Number of professional council:

7) Area of professional practice:
   - Physician working in emergency room
   - Physician working in intensive care unit
   - Physician working in clinic
   - Nurse working in intensive care unit
   - Nurse working in ward
   - Physiotherapist working in intensive care unit
   - Physiotherapist working in ward
   - Nutritionist working in intensive care unit
   - Nutritionist working in ward
   - Other

8) Are you providing direct care to patients with COVID-19 diagnosis?
   - Yes
   - No

9) Are you felling or have you felt any of these symptoms in the last days?
   - Fever
   - Dry cough
   - Secretive cough
   - Difficulty to breath
   - Tiredness

- Muscle pain
- Rhinorrhea
- Sore throat
- Nasal blockage
- Abdominal pain / discomfort
- Nausea or vomiting
- Loss or reduction of smell
- Loss or reduction of taste
- Burning within the nose
- Other symptoms
- No symptom

10) Detail the others symptoms:

11) If you felt loss or reduction of smell:
- This symptom occurred before the symptoms of item 9
- This symptom occurred concomitant to the symptoms of item 9
- This symptom occurred after the symptoms of item 9
- I did not lose sense of smell

12) If you felt loss or reduction of taste:
- This symptom occurred before the symptoms of item 9
- This symptom occurred concomitant to the symptoms of item 9
- This symptom occurred after the symptoms of item 9
- I did not lose taste

13) If you felt loss or reduction of smell:
- I lost for 1 to 5 days, but I have already recovered it
- I lost for 5 to 10 days, but I have already recovered it
- I lost for 10 to 15 days, but I have already recovered it
- I lost for more than 15 days, but I have already recovered it
- I lost for 1 to 5 days and I did not recover it yet
- I lost for 5 to 10 days and I did not recover it yet
- I lost for 10 to 15 days and I did not recover it yet
- I lost for more than 15 days and I did not recover it yet
- I did not lose sense of smell

14) If you felt loss or reduction of taste:
- I lost for 1 to 5 days, but I have already recovered it
- I lost for 5 to 10 days, but I have already recovered it
- I lost for 10 to 15 days, but I have already recovered it
- I lost for more than 15 days, but I have already recovered it
- I lost for 1 to 5 days and I did not recover it yet
- I lost for 5 to 10 days and I did not recover it yet
- I lost for 10 to 15 days and I did not recover it yet
- I lost for more than 15 days and I did not recover it yet
- I did not lose taste

15) Did you have any difficulty to smell things or to taste food before presenting the current

- Yes
- No

16) Do you have or have recently suffered any of the following conditions?

- Neurodegenerative disease (Alzheimer, Parkinson, Multiple sclerosis)
- Epilepsy
- Stroke
- Brain tumor
- Arms or legs paralysis
- Severe memory problem
- Chronic sinusitis
- Tumor within the nose
- Nose bleeding
- Current or previous smoking
- Use of heart or psychiatric medication
- Fall or head trauma leading to loss of consciousness
- None of the items above

17) Have you been tested for COVID-19?

- Yes, I have, RT-PCR and the result was negative
- Yes, I have, anti-COVID-19 IgG/IgM rapid test and the result was negative
- Yes, I have, RT-PCR and the result was positive
- Yes, I have, anti-COVID-19 IgG/IgM rapid test and the result was positive
- No, I have not been tested
